# New protein–protein interactions of mitochondrial connexin 43 in mouse heart

**DOI:** 10.1111/jcmm.12792

**Published:** 2016-02-25

**Authors:** Amanda Denuc, Estefanía Núñez, Enrique Calvo, Marta Loureiro, Elisabet Miro‐Casas, Adela Guarás, Jesús Vázquez, David Garcia‐Dorado

**Affiliations:** ^1^ Vall d'Hebron University Hospital and Reseach InstituteUniversitat Autònoma de BarcelonaBarcelonaSpain; ^2^ Centro Nacional de Investigaciones Cardiovasculares Carlos IIIMadridSpain

**Keywords:** connexin 43, apoptosis‐inducing factor, electron‐transfer protein, mitochondria, cardiomyocyte

## Abstract

Connexin 43 (Cx43), the gap junction protein involved in cell‐to‐cell coupling in the heart, is also present in the subsarcolemmal fraction of cardiomyocyte mitochondria. It has been described to regulate mitochondrial potassium influx and respiration and to be important for ischaemic preconditioning protection, although the molecular effectors involved are not fully characterized. In this study, we looked for potential partners of mitochondrial Cx43 in an attempt to identify new molecular pathways for cardioprotection. Mass spectrometry analysis of native immunoprecipitated mitochondrial extracts showed that Cx43 interacts with several proteins related with mitochondrial function and metabolism. Among them, we selected for further analysis only those present in the subsarcolemmal mitochondrial fraction and known to be related with the respiratory chain. Apoptosis‐inducing factor (AIF) and the beta‐subunit of the electron‐transfer protein (ETFB), two proteins unrelated to date with Cx43, fulfilled these conditions, and their interaction with Cx43 was proven by direct and reverse co‐immunoprecipitation. Furthermore, a previously unknown molecular interaction between AIF and ETFB was established, and protein content and sub‐cellular localization appeared to be independent from the presence of Cx43. Our results identify new protein–protein interactions between AIF‐Cx43, ETFB‐Cx43 and AIF‐ETFB as possible players in the regulation of the mitochondrial redox state.

## Introduction

Connexin 43 (Cx43) is the predominant connexin isoform in ventricular myocardium. It is widely known to participate in cell‐to‐cell propagation of the electrical impulse as well as in chemical communication between adjacent cells. As a pore‐forming molecule, it is composed of four transmembrane domains as well as intracellular amino‐ and carboxyterminal domains 1. Connexins are assembled in groups of six units to form hemichannels, or connexons, that may come into contact with a hemichannel from a neighbouring cell to form a gap junction (GJ) channel. These intercellular channels conform a specialized membrane area of chemical communication that usually integrates other adhesion structures, such as desmosomes or adherens junctions 2. Importantly, channel activity is determined by the conduction properties of the connexin involved. Thus, Cx43 connexons have a minimal diameter pore of 15 Å, with a relative anion‐to‐cation selectivity ratio of 1.17, and an intermediate unitary conductance of 100 pS 3, 4, 5.

Besides their role in the formation of permeable transmembrane channels ensuring electrical and metabolic coupling between cells, Cx43 has been shown to participate in several signalling pathways 6, 7. Normal sodium and potassium currents appear to be dependent on Cx43 expression 8, 9, and therefore, any alteration of this protein can modify the action potential of cardiomyocytes with arrhythmogenic consequences. Connexins may also control cell growth and inhibit tumorigenicity in different cell types; it has been proved that overexpression of Cx43 in neonatal rat cardiomyocytes decreases DNA synthesis 10. In Cx43‐deficient mice, embryonic development is compromised and animals die at birth due to swelling and blockage of the right ventricular outflow tract from the heart 11. At the connexon level, several mechanisms nonrelated to GJ communication have been proposed to modulate cell death and survival, paracrine factors release, volume regulation or activation of signal transduction pathways 12. Although the end effectors of these mechanisms remain poorly established, a complete understanding of connexin function has been achieved when defining its molecular interactors, like kinases, phosphatases or other molecules regulated by receptor stimulation or subcellular localization 13.

Furthermore, Cx43 is involved in myocardial energy metabolism, tolerance to ischaemia and preconditioning protection—*i.e*. protection against cell death obtained by brief non‐lethal episodes of ischaemia preceding prolonged severe ischaemia 14, 15. Most of the signalling pathways of ischaemic preconditioning converge on mitochondria, and recent reports have shown that in cardiomyocytes Cx43 is also located at the inner membrane of subsarcolemmal mitochondria (SSM), whereas it is nearly absent at interfibrillar mitochondria (IFM) 16, 17. *In vitro* cross‐linking studies show mitochondrial complexes with a molecular weight comparable to that of sarcolemmal Cx43 hemichannels; in addition, those complexes are sensitive to carbenoxolone and heptanol, two hemichannel blockers 18. It has also been described that mitochondrial Cx43 (mtCx43) modulates mitochondrial potassium influx and respiration 18, 19. Moreover, in the presence of mtCx43, ischaemic preconditioning prevents reperfusion‐induced respiratory failure and oxidative damage, whereas lack of mtCx43 abolishes the protective effect of preconditioning on myocardial infarct size 15, 20, 21. However, the molecular effectors of the cardioprotective role of Cx43 still remain elusive. Mitochondrial Cx43 could regulate mitochondrial volume and, as a consequence, respiration and reactive oxygen species (ROS) signalling by forming hemichannels similar to those present at the sarcolemma. Alternatively, mtCx43 could regulate mitochondrial function and cardioprotection by interacting with still unknown proteins.

The aim of this study is to find new molecular partners of mtCx43 that could participate in putative channel‐independent actions. Proteomics analysis identifies apoptosis‐inducing factor (AIF) and the beta‐subunit of the electron‐transfer protein (ETFB), two unrelated proteins involved in oxidative phosphorylation and redox control, as molecular interactors of mtCx43. Our results also confirm that AIF and ETFB are able to interact in a Cx43‐independent manner.

## Materials and methods

### Mice

Adult male and female transgenic Cx43KI32 mice (developed by 22) aged between 16 and 20 weeks (30 g) were used in this study. In these animals, the coding region of the *Cx43* gene has been replaced by that of *Cx32* as confirmed by specific PCR analysis. All protocols were approved by the Ethics Committee on Animal Research of the Hospital Vall d'Hebron, Barcelona, Spain. The study conforms the European Directive on the Welfare of Research Animals (2010/63/UE).

### Mitochondrial and total heart extracts

Mice heart mitochondria were prepared by differential centrifugation 18, 23. Briefly, for each pull down assay, 6–8 animals from both wild‐type (Cx43 WT; Cx43 homozygous genotype) and knock‐in mutant mice (Cx43 KI Cx32; Cx32 homozygous genotype), considered two separate groups, were deeply anaesthetized by an i.p. injection of sodium pentobarbital (150 mg/kg). Whole hearts were quickly excised, minced and homogenized with 5–10 strokes of a teflon pistill in a glass potter at 250 r.p.m. in ice‐cold isolation buffer A [in mmol/L: 290 sucrose, 5 3‐(N‐Morpholino)‐propanesulfonic acid (MOPS), 2 ethylene glycol‐bis(2‐aminoethylether)‐*N*,*N*,*N*′,*N*′‐tetraacetic acid (EGTA), 10 NaF, 2 Na_3_VO_4_ and protease inhibitors cocktail (P8340; Sigma‐Aldrich, Saint Louis, MO, USA), pH 7.4]. Samples were centrifuged at 750 g and supernatant obtained handled as the subsarcolemmal mitochondrial fraction (SSM). The sediment of this first centrifugation was further processed with 2 mg proteinase K (P2308; Sigma‐Aldrich)/g heart tissue in ice‐cold isolation buffer B [in mmol/L: 100 KCl, 50 MOPS, 2 EGTA, and 10% bovine serum albumin (BSA), pH 7.4] to obtain the interfibrillar mitochondrial fraction (IFM). Two additional centrifugation steps are necessary for mitochondrial purification. The first one at 5000 × g to obtain an enriched SSM and IFM fraction, followed by a second centrifugation at 12,500 × g in 17% percoll in isolation buffer A. Total heart homogenates were attained by solubilization of 100–200 mg of tissue in lysis buffer [2% *n*‐Dodecyl β‐d‐maltoside (D4641; Sigma‐Aldrich), 20 mmol/L TEA pH 8, 20 mmol/L ethylenediaminetetraacetic acid, 10 mmol/L DTT and protease inhibitors cocktail]. All samples were kept on ice for immediate use, and protein concentration was determined by Bradford assay.

### Co‐Immunoprecipitation experiments

A total of 0.8–1 mg of protein was used for each pull down reaction with 10 μg of rabbit anti‐connexin‐43 antibody (C6219; Sigma‐Aldrich), mouse anti‐apoptosis‐inducing factor antibody (MA5‐15880; Pierce‐Thermo Scientific, Rockford, IL, USA), rabbit anti‐ETFB subunit beta antibody (17925‐1‐AP; ProteinTech, Manchester, UK), respectively, according the Pierce^®^ Crosslink Immunoprecipitation Kit manufacturer's instructions (26147; Thermo Scientific, Rockford, IL, USA). Total and mitochondrial heart extracts were treated in non‐denaturing conditions with buffers supplied, and protein‐antibody‐agarose beads complexes incubated at 4°C during 16 hrs with an end‐over‐end mixing. After washing, bound proteins were eluted (low pH) and preserved for posterior proteomics analysis and Western blotting.

### Proteomics

#### Protein digestion

The resulting soluble protein extracts were run on a SDS‐PAGE gel (10% resolving gel and 4% stacking gel) at 50 V. The electrophoresis was stopped when the front dye has barely passed into the resolving gel, ensuring concentration of all proteins into a unique band. Staining was performed using GelCode^®^ Blue Stain Reagent (Thermo Scientific). Gel pieces were cut into cubes (2 mm). For the protein digestion, modified porcine trypsin (Promega, Fitchburg, WI, USA) was added at a final ratio of 1:20 (trypsin‐protein). Digestion proceeded overnight at 37°C in 100 mM ammonium bicarbonate, pH 8.8.

#### Identification of proteins by liquid chromatography coupled to tandem mass spectrometry

The resulting tryptic peptide mixtures were subjected to nano‐liquid chromatography coupled to mass spectrometry (LC‐MS) for protein identification. Peptides were injected onto a C‐18 reversed phase (RP) nano‐column (75 μm I.D. and 50 cm, Acclaim PepMap100; Thermo Scientific) and analysed in a continuous acetonitrile gradient consisting of 0–30% B in 240 min. and 50–90% B in 3 min. (B = 90% acetonitrile, 0.5% acetic acid). A flow rate of ca. 200 nl/min. was used to elute peptides from the RP nano‐column to an emitter nanospray needle for real‐time ionization and peptide fragmentation on a Orbitrap Elite mass spectrometer (Thermo Fisher, San José, CA, USA). An enhanced FT‐resolution spectrum (resolution = 120,000) followed by the MS/MS spectra from most intense fifteen parent ions was analysed along the chromatographic run (272 min.). Dynamic exclusion was set at 30 sec. For protein identification, tandem mass spectra were extracted and charge state deconvoluted by Proteome Discoverer 1.4.0.288 (Thermo Fisher Scientific). All MS/MS samples were analysed using SEQUESTTM (Thermo Fisher Scientific).

From an initial list of 493 identified proteins, 78 were proposed as potential candidates according to the different proportion of peptides detected between the SSM and IFM sample, favouring the former. Among this list of 78, eight candidates were finally chosen according to the following criteria (see Table S1 for full list): (*i*) cytoskeleton proteins were excluded, as we were working mitochondrial specificity; (*ii*) proteins present in the IFM sample were excluded, as Cx43 is not represented in this fraction; and finally, (*iii*) proteins were chosen according to the bibliographic data available related with the functional capacity of the organelle (UniProt Database, UniProt Consortium).

#### Blue‐native electrophoresis and MS distribution analyses

Mitochondria‐enriched samples from three different tissues (liver, heart and brain) and from a fibroblasts cell line (fC57) were treated with 4% digitonin to solubilize membrane proteins. Blue‐native electrophoresis (BNE) was used to separate the native complexes in a gradient gel from 4% to 13% acrylamide. Staining was performed using GelCode^®^ Blue Stain Reagent (Thermo Scientific). Each lane was divided and excised in 26 bands covering the whole electrophoretic run. Gel pieces were then digested with trypsin following the aforementioned protocol, and the resulting tryptic peptide mixtures were subjected to LC‐MS analysis for identifying the proteins present on each band.

### Suborganelle protein localization

To assess the mitochondrial localization of the different proteins of interest, 100–300 μg of SSM protein, either from wild‐type or knock‐in mutant mice, was incubated for 10 min. on ice with different proteinase K concentrations (0.1–0.5 μg/ml; Sigma‐Aldrich), in order to partially digest the outer membrane. Samples were further analysed by Western blotting.

### Western blot

Protein samples (25–30 μg of total protein/lane) were mixed 1:1 with 2× protein loading buffer (60 mmol/L Tris‐HCl pH 6.8, 10% glycerol, 2% SDS, 1% bromophenol blue, 10% beta‐mercaptoethanol), boiled 5 min. at 98°C and loaded onto 12% SDS‐PAGE gels. Proteins were detected by Western blotting using: rabbit anti‐Cx43 antibody (C6219; Sigma‐Aldrich), mouse anti‐AIF antibody (MA5‐15880; Pierce‐Thermo Scientific), rabbit anti‐ETFB antibody (17925‐1‐AP; ProteinTech), mouse anti‐Na^+/^K^+^ ATPase alpha‐1 antibody (05‐369; Millipore, Billerica, MA, USA), mouse anti‐SDHA (MS204; MitoSciences, Eugene, OR, USA), mouse anti‐Pan Cadherin (C1821; Sigma‐Aldrich) and rabbit anti‐TOMM20 antibody (HPA011562; Sigma‐Aldrich).

### Immunofluorescent assays

#### Isolation of adult cardiac myocytes

Adult cardiac cells from either wild‐type or knock‐in Cx43 mice were obtained by retrograde collagenase perfusion 15. Briefly, adult mice were deeply anaesthetized by an i.p. injection of sodium pentobarbital (150 mg/kg), and the heart was perfused in a Langendorff system for 15 min. with a modified Krebs buffer with 0.03% type II collagenase (17456; Serva Electrophoresis, Heidelberg, Germany). Calcium‐tolerant rod‐shaped cardiomyocytes were selected by gravity sedimentation in 4% BSA gradient and plated on laminin‐coated cover slips.

#### Subcellular localization assays

Isolated cardiomyocytes were fixed and permeabilized with ice‐cold acetone during 8 min. at RT. After washing with PBS + 0.025% Triton X‐100, samples were blocked with 1% BSA in PBS for 45 min. at RT and immunodetected using rabbit anti‐Cx43 antibody (C6219; Sigma‐Aldrich), mouse anti‐AIF antibody (MA5‐15880; Pierce‐Thermo Scientific), rabbit anti‐ETFB antibody (17925‐1‐AP; ProteinTech), mouse anti‐SDHA (MS204; MitoSciences) and rabbit anti‐VDAC antibody (ab15895; AbCam, Cambridge, UK) as primary antibodies and AlexaFluor 488‐conjugated antimouse (A‐11001; Molecular Probes, Thermo Scientific, Rockford, IL, USA) and AlexaFluor 594‐conjugated anti‐rabbit (A‐11012; Molecular Probes) as secondary antibodies. Finally, cardiomyocytes were washed twice with PBS and nuclei were stained with Hoeschst 33342 (10 μg/ml). Confocal fluorescent images were captured with a spectral microscope (Olympus Spectral Confocal Microscopy FV1000, Olympus, Center Valley, PA, USA). Images were captured taken into account that the pixel size was set to 1/2 of the optical resolution, and quantitative data were obtained with ImageJ software analysis (JACoP plug‐in) 24.

#### Proximity ligation assay

Cardiomyocytes from either wild‐type or knock‐in Cx43 mice were isolated, plated in cover slips and incubated with the corresponding primary antibodies as previously described. Preparations were then tested using the DuoLink kit secondary antibodies conjugated with the corresponding proximity ligation assay (PLA) probes (DUO92101; Sigma‐Aldrich) for 1 hr 37°C. For ligation and amplification reactions, DuoLink *in situ* detection kit recommendations were followed. Cell images were obtained with spectral microscope (Olympus Spectral Confocal Microscopy FV1000) as stated above. Positive cross‐reactivity was quantified in background‐subtracted images using BlobFinder software (developed by: Carolina Wählby and Amin Allalou at CBA, Uppsala University) 25.

### Statistics

Data are expressed as mean ± S.E.M. Student's *t*‐test was used to compare Manders' coefficients and PLA quantification values between Cx43 WT and Cx43 KI Cx32 transgenic animals. Differences were considered significant when *P* < 0.05.

## Results

### Mitochondrial Cx43 interacts with proteins related with the respiratory electron chain

To examine new molecular partners of Cx43, a first set of immunoprecipitation experiments was done in non‐denaturing conditions to preserve and analyse potential protein–protein interactions. Cardiac subsarcolemmal (SSM) and interfibrillar (IFM) mitochondrial extracts were purified from Cx43 WT mice hearts and 1 mg of total protein was processed with a specific anti‐Cx43 antibody. IFM elution sample obtained was used as a negative control, as Cx43 is virtually absent in this mitochondrial population 17. Protein content of the eluted fractions was analysed by mass spectrometry and final results are summarized in Table 1.

**Table 1:** Eluted samples from three independent Cx43 immunoprecipitation experiments were analysed and the most regular results were selected from a total of 80 candidates. Gene, UniProt code and complete name are shown, and the biological processes known indicated

Gene	Protein	UniProt	Protein name	Biological process
Gja1	Cx43	P23242	Gap junction alpha‐1 protein	Cx43 ‐ gap junction channel
Eci1	ECI1	P42125	Enoyl‐CoA delta isomerase 1	Fatty acid beta‐oxidation
Pfkm	PFKM	P47857	ATP‐dependent 6‐phosphofructokinase	Carbohydrate degradation, glycolysis
Ndufs6	NDUS6	P52503	NADH dehydrogenase [ubiquinone] iron‐sulphur protein 6	Electron transport, respiratory chain
Echs1	ECHS1	Q8BH95	Enoyl‐CoA hydratase	Fatty acid beta‐oxidation
Acat1	ACAT1	Q8QZT1	Acetyl‐CoA acetyltransferase	Ketone body metabolism
Cs	CS	Q9CZU6	Citrate synthase	Tricarboxylic acid cycle
Etfb	ETFB	Q9DCW4	Electron‐transfer flavoprotein subunit beta	Electron transport, electron carrier
Aifm1	AIF	Q9Z0X1	Apoptosis‐inducing factor 1	Apoptosis, cell redox homeostasis, respiratory chain complex I assembly

To sum up, from an initial list of 78 protein candidates, eight were finally chosen based on its involvement in mitochondria function. When candidates were examined focusing on its main biological function, three of them had special interest for being associated to mitochondrial respiration, as Cx43 is described to have an impact on respiratory complex I activity and oxygen consumption 19. *Ndufs6* is an accessory subunit of the mitochondrial membrane respiratory chain NADH dehydrogenase (Complex I) that is believed not to be involved in catalysis; *Etfb*, in association with *E*tfa, acts as an electron carrier from fatty acid beta‐oxidation to the electron transport chain *via* ETF‐ubiquinone oxidoreductase; and finally, *Aifm1* is mostly known as a caspase‐independent apoptogenic molecule but, in the absence of specific death signals, it has a NADH oxidoreductase activity (biological process extracted from UniProt Database).

### Mitochondrial Cx43 co‐immunoprecipitates with AIF and ETFB

**Figure 1:**
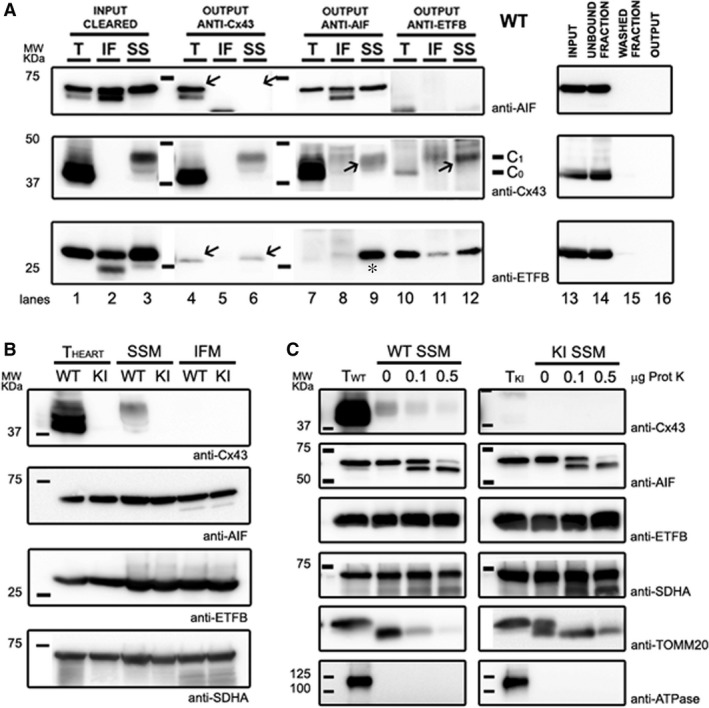
Western Blot analysis of protein co‐immunoprecipitation and mitochondrial distribution of Cx43, ETFB and AIF in WT and Cx43KICx32 mice. (**A**) A total amount of 1 mg of heart mitochondrial protein extracts from WT mice was immunoprecipitated using anti‐Cx43, anti‐AIF and anti‐ETFB antibodies. In left panels, lanes from 1 to 3 correspond to the input sample, the initial total (T), subsarcolemmal (SSM) or interfibrillar (IFM) protein extracts. Lanes from 4 to 12 correspond to the output sample—the immunoprecipitated fraction—analysed for the presence of each protein. A representative IgG control of Cx43‐IP protocol is shown in right panels using the total protein extract as a sample. Of note, the Cx43 antibody used recognizes also the phosphorylated protein forms (C_0_ unmodified, C_1_ phosphorylated). Also, due to proteinase treatment to obtain the IFM fraction, AIF is partially truncated and noted as a double lower molecular weight band. (**B**) Protein content of Cx43, AIF and ETFB. Anti‐SDHA was used as a mitochondrial loading control. (**C**) Sub‐mitochondrial distribution of Cx43, AIF and ETFB proteins was examined after partial digestion of the outer mitochondrial membrane with 0.1 and 0.5 μg of proteinase K, as indicated. Na^+^/K^+^ ATPase reactivity was assessed to rule out any potential contamination from gap‐junctional membranes, and SDHA and TOMM20 were used as an inner and outer mitochondrial membrane protein control, respectively. A representative image of at least three independent replicates is shown.

Because no previous interaction has been described so far for these proteins, we next proceeded to confirm the proteomics results by Western blot analysis of the immunoprecipitated samples (Fig. 1A). For this purpose, we used purified SSM mitochondrial extracts from Cx43 WT mice, including the IFM fraction as a negative control. A good recovery of endogenous Cx43 was achieved, and a faint band was detected in the eluted samples, both for ETFB and AIF proteins (Fig. 1A, lanes 4–6 arrows), although AIF content on SSM Cx43‐immunoprecipitated sample was barely perceptible. In addition, when performing reverse co‐immunoprecipitation to analyse the presence of Cx43 in the anti‐ETFB and anti‐AIF eluted samples, Cx43 was present in both mitochondrial extracts, most notably in the SSM fraction, validating the proteomics results (Fig. 1A, lanes 7–12 arrows).

We were unable to prove any co‐immunoprecipitation of Cx43 with NDUFS6 (data not shown), and so it was discarded from our study.

Remarkably, the AIF‐immunoprecipitated fraction showed reactivity against ETFB, pointing to a new protein interaction between these two proteins (Fig. 1A, lane 9 asterisk).

### Mitochondrial protein amount or sub‐organelle distribution of ETFB and AIF is not dependent on the presence of Cx43

Evidence from the immunoprecipitation assay prompted us to investigate if Cx43 content could either modulate protein amount or sub‐localization of ETFB and AIF in mitochondria. ETFB is supposed to remain in the mitochondrial matrix, and AIF displays an inner membrane‐anchored mature form and an intermembrane‐matrix‐soluble form just after a proteolytic stimulus 26. We first studied the total protein amount in whole heart samples and in SSM and IFM fractions from Cx43 WT and Cx43 KI Cx32 animals, respectively (Fig. 1B). No significant differences in protein content were found between both genotypes.

When exploring the mitochondrial distribution, 1–300 μg of SSM protein extract were digested with 0.1 and 0.5 μg of proteinase K to eliminate the outer mitochondrial membrane and expose the intermembrane matrix and the inner mitochondrial membrane (Fig. 1C). AIF was sensitive to proteinase K action, and proteolysis of the anchored mature form was detected as a double band of lower molecular weight (Fig. 1C lanes 3 and 4). Overall, no statistically significant differences in protein pattern were found related to Cx43 presence, suggesting that sub‐organelle localization of AIF or ETFB does not depend on their interaction with Cx43.

### Sub‐cellular localization of Cx43, ETFB and AIF in cardiomyocyte mitochondria

**Figure 2:**
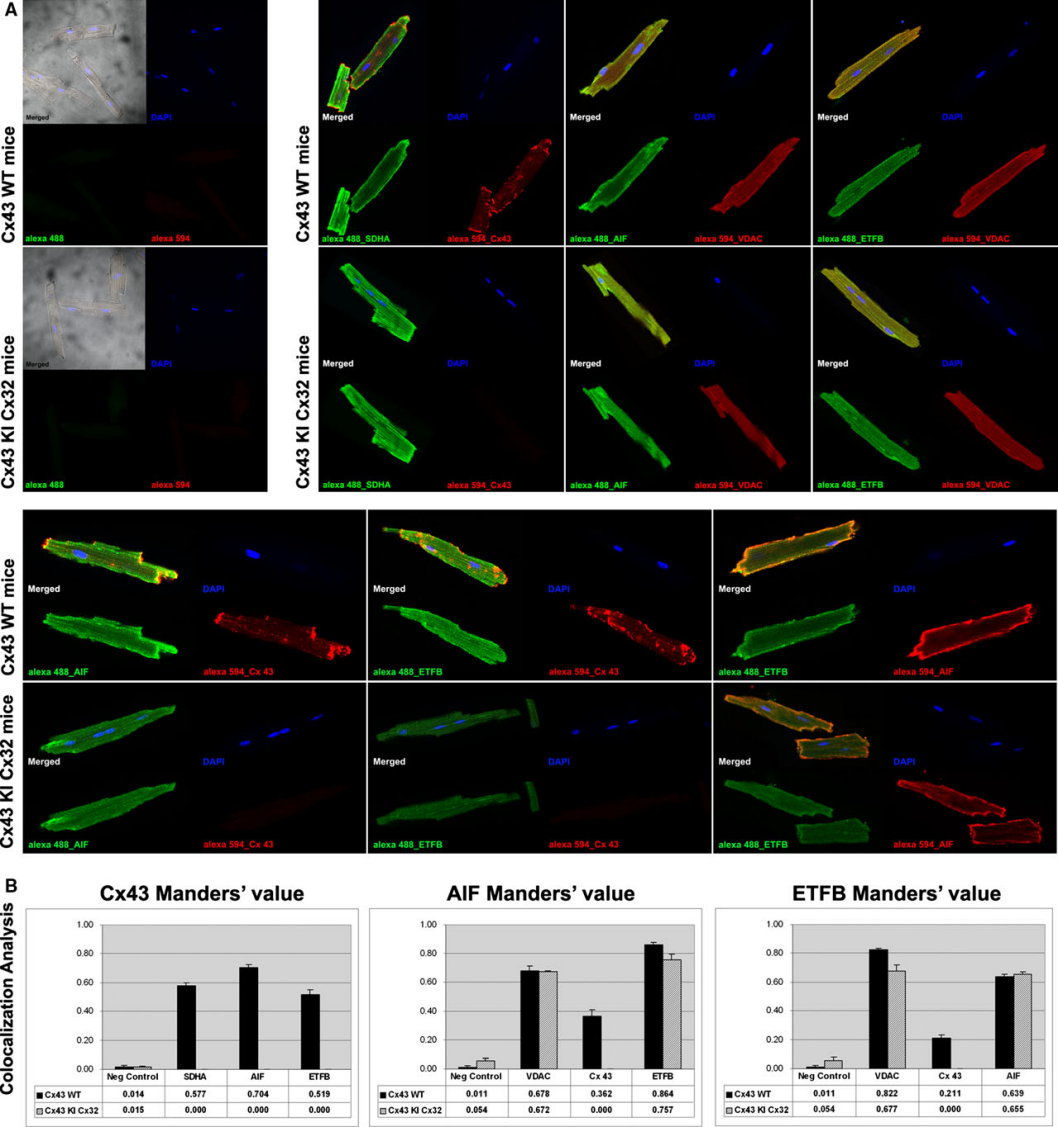
Immunolocalization of Cx43, ETFB and AIF proteins in freshly isolated cardiomyocytes from WT and Cx43KICx32 mice. (**A**) Confocal microscopy images showed a specific localization in cardiomyocyte membrane and mitochondria for Cx43; and presence in mitochondria for AIF and ETFB, in agreement with bibliographic information (upper right snapshots). Co‐localization (lower snapshots) of Cx43 with AIF or ETFB, and also AIF with ETFB, is quantitatively evaluated in (**B**) by analysis of composed images using 8–12 z‐stacks for each experimental condition, of at least three independent cardiomyocyte isolation events for each genotype. See text for comment on Manders' coefficients.

We next investigated if mitochondrial Cx43 is coincident with sub‐cellular localization of AIF and ETFB (Fig. 2). For this purpose, adult isolated cardiomyocytes from both Cx43 WT or Cx43 KI Cx32 mice were immunolabelled with anti‐AIF, anti‐ETFB or anti‐Cx43. Positive labelling of mitochondrial proteins SDHA and VDAC were used as control (Fig. 2A). Manders' coefficients M1 and M2 were quantified with ImageJ software (Fig. 2B). On the whole, 51.9 ± 3.3% of Cx43 labelling co‐localized with ETFB, and 70.4 ± 2.3% with AIF, results that suggest a strongest co‐localization with AIF protein in the mitochondria. Manders' coefficient between ETFB and AIF labelling was 63.9 ± 1.6% in WT *versus* 65.5 ± 1.3% in KI mice. Manders' coefficient between AIF and ETFB labelling was 86.4 ± 1.6% in WT *versus* 75.7 ± 3.8% in KI mice. Again, no statistically significant differences were detected between different genotypes, suggesting a Cx43‐independent interaction.

### Interaction of Cx43, ETFB and AIF in cardiomyocyte mitochondria

**Figure 3:**
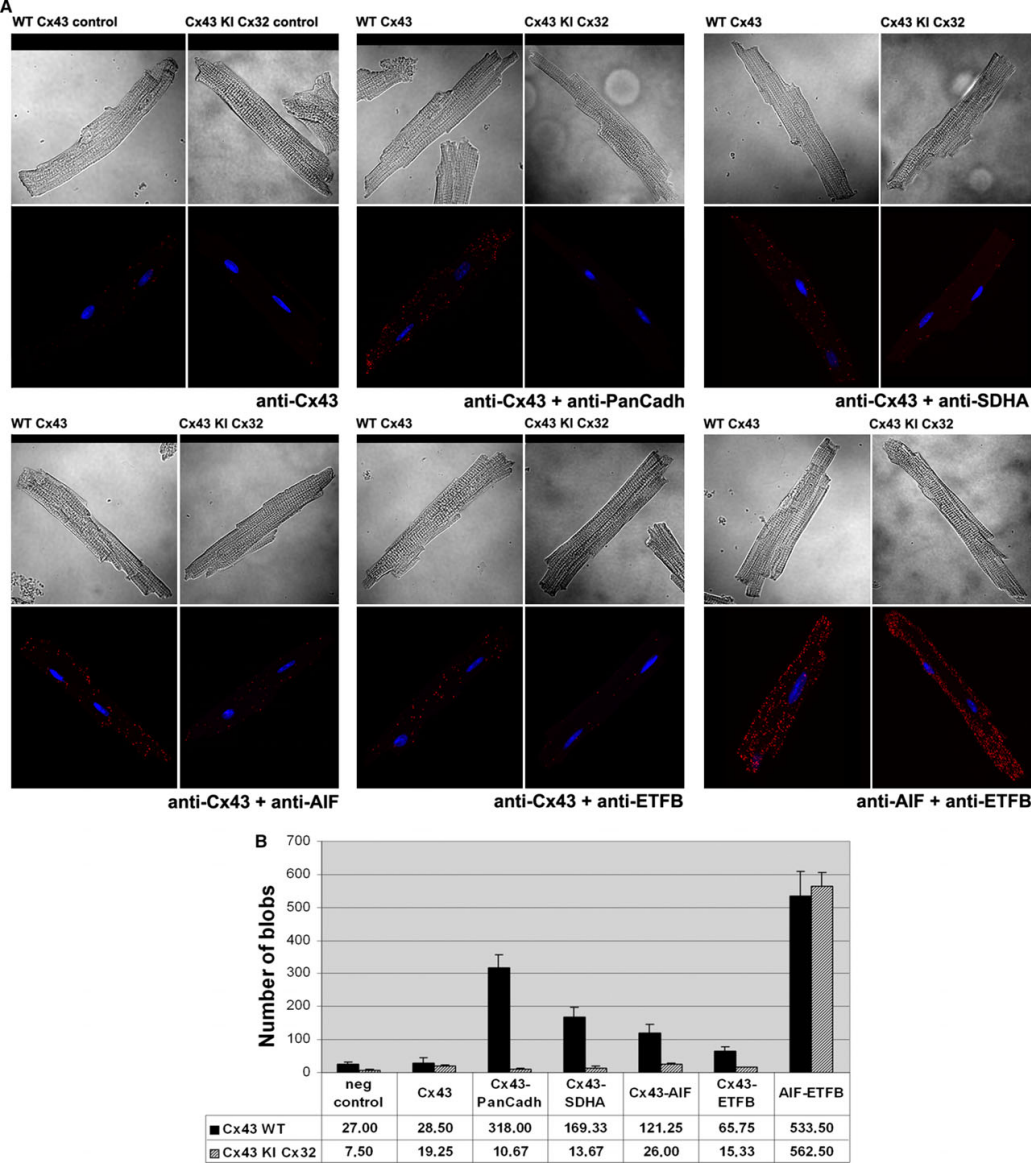
*In situ* proximity ligation assay of Cx43, ETFB and AIF proteins in freshly isolated cardiomyocytes from WT and Cx43KICx32 mice. (**A**) Cells treated with no primary antibody (negative control) or with only anti‐Cx43 were used to determine the signal specificity, whereas Cx43‐PanCadherin and Cx43‐SDHA pairs of proteins were used as a positive control. In the lower group of confocal images, Cx43 interacts with AIF and ETFB, as well as AIF with ETFB in a Cx43‐independent manner. (**B**) Assessment of the number of signals detected with composed images using 8–12 z‐stacks for each experimental condition, of at least three independent cardiomyocyte isolation events for each genotype.

To quantify the possible interaction between our proteins of interest, we used an *in situ* PLA, in which a pair of labelled secondary antibodies generates a fluorescent signal only if they are in close proximity (<40 nm), either in a stable or a transient interaction, being the latter a possible explanation for the faint levels detected with co‐immunoprecipitation assays. As shown in Figure 3, confocal images obtained after PLA of freshly isolated adult cardiomyocytes demonstrated fluorescent cross‐reactivity between Cx43‐AIF and Cx43‐ETFB (Fig. 3A). The number of positive fluorescent spots was 121.25 ± 25.10 and 65.75 ± 10.89 for Cx43‐AIF and Cx43‐ETFB pairs, respectively.

Apoptosis‐inducing factor‐ETFB protein–protein interaction was proved to be persistent and not depending on Cx43, as quantification of the number of fluorescent spots revealed no statistically significant differences between genotypes: 533.5 ± 76.82 in Cx43 WT cardiomyocytes *versus* 562.5 ± 42.66 in Cx43 KI Cx32 cardiomyocytes (Fig. 3B).

### AIF and ETFB comigrate in blue‐native gels

**Figure 4:**
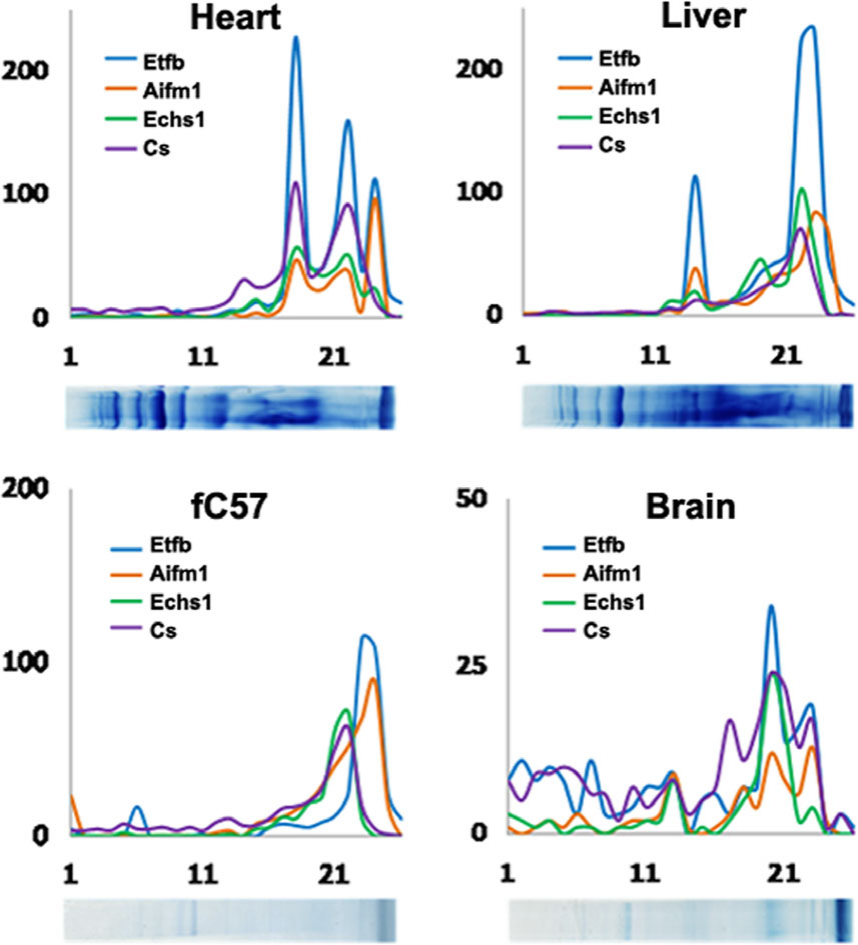
AIF, ETFB, ECHS1 and CS protein patterns in blue‐native gels. High correlation in the distribution patterns for proteins AIF, ETFB, ECHS1 and CS is showed according with their BNE mobility in heart, liver, brain and fC57 the cell line.

The possible interaction between AIF and ETFB was further characterized by blue‐native gel electrophoresis followed by mass spectrometry analysis of each fraction from heart, liver and brain tissues and fC57 liver cell line. We found that AIF and ETFB had the same migration profile in the four samples analysed, suggesting the possible interaction and formation of the same macromolecular complex of these proteins (Fig. 4). Moreover, the molecular weight that corresponds to the bands where the two proteins are identified is higher than the molecular weight of the individual proteins. In addition, we found that enoyl‐CoA hydratase (ECHS1), a protein implicated in fatty acid beta‐oxidation, and citrate synthase (CS), implicated in tricarboxylic acid cycle, displayed also the same migration pattern as ETFB and AIF in the three tissues analysed (Fig. 4). All together, these results reinforce our data and are in agreement with previous studies that describe the association between fatty acid oxidation enzymes and respiratory complexes (see Discussion).

## Discussion

In this study, we investigated the interaction of Cx43 with new molecular partners in mitochondria from adult mouse cardiomyocytes under non‐stress conditions. By using co‐immunoprecipitation assays and second‐generation proteomics, we provide evidence that mtCx43 interacts with AIF and ETFB, two unrelated proteins to date. This interaction was further supported by colocalization and comigration studies that also demonstrate a previously unsuspected protein–protein interaction between AIF and ETFB. However, this same interplay found in mitochondria from transgenic animals lacking Cx43 suggests that these proteins may interact independently from mtCx43, most probably within a multi‐protein complex. These results may serve as a starting point for the investigation of new aspects on the regulation of mitochondrial function. The identification of Cx43 interactors has important practical implications as it can help to elucidate the molecular pathway of ischaemic preconditioning cardioprotection and to propose potential pharmacological targets for the development of new therapeutic strategies.

Apoptosis‐inducing factor is an evolutionarily conserved flavoprotein, known as a cell death‐promoting molecule after proapoptotic signals. Nevertheless, under physiological conditions, AIF is involved in oxidative phosphorylation and redox management 26, 27. Data gathered from independent studies reveal that AIF‐knockout cells and tissue‐specific knockouts of the *Aifm1* gene in the heart and the skeletal muscle exhibit a reduction in respiratory complex I protein content or/and activity 28, 29. Most interestingly, deficient AIF mice develop more severe heart injury when subjected to myocardial ischaemia‐reperfusion, an effect that has been related to a reduced capacity of SSM to scavenge free radicals 30. Importantly, these evidences are in agreement with functional data on mitochondrial complex I activity and ROS handling in previous studies with Cx43‐deficient mice 19, 20.

Electron‐transfer flavoprotein is a mitochondrial matrix heterodimer with alpha‐ (ETFA, 30 kD) and beta‐ (ETFB, 28 kD) subunits that contains one FAD and one AMP molecule per heterodimer 31. ETF and ETF‐ubiquinone oxidoreductase are the two enzymes required for electron transfer from at least 11 dehydrogenases in the fatty acid beta‐oxidation cascade to the mitochondrial respiratory chain. However, very few studies have explored the role of ETF or any of its subunits on mitochondrial respiratory function and ROS generation. In a recent study, Rodrigues and Gomes 32 analysed the effect of two pathogenic point mutations in human ETFB—which are believed to prevent its interaction with the FAD cofactor and its partner dehydrogenases—on superoxide and hydrogen peroxide generation. According to their results, wild‐type ETF is able to produce significant amounts of both superoxide and hydrogen peroxide, but this reactivity is decreased when forming part of a tight protein–protein complex. The capacity to form such protein complexes depends on ETFB subunit, as disease mutants show higher production of ROS. In agreement with a multi‐protein scenario, other studies have shown that fatty acid oxidation enzymes and respiratory chain complexes are physically associated, comprising multifunctional electron‐transfer chain complexes 33.

Our data provide a new lead about the management of the redox status in cardiomyocyte mitochondria showing that mtCx43 interacts preferentially with AIF and also with ETF—through its beta‐subunit. In the context of available data on the roles of these proteins in the modulation of mitochondrial respiration and ROS production, these results may indicate the participation of mtCx43, ETF and AIF in multi‐protein complexes with important actions on mitochondrial physiology. Reactive oxygen species and mitochondrial function are strongly interconnected both in normal and dysfunctional states. The concentration of these reactive species shifts the overall redox balance between their role as signalling molecules to their role in promoting deleterious effects. The control of this balance may depend on various interacting proteins working together, most probably in localized dynamic platforms.

Further studies will be necessary to determine the composition and functions of these putative multi‐protein complexes, and in particular whether its formation may explain the important modulatory effect of mtCx43 on mitochondrial respiration and ROS production. Our present data indicate that mtCx43 may not be an indispensable element in these complexes, as its absence does not appear to modify the cellular localization of ETFB or AIF, nor the interaction between them.

## Conclusion

The present study proposes the interaction of mtCx43 with ETFB and AIF to form multi‐protein complexes with effects on mitochondrial respiration and ROS signalling. Elucidation of the composition and functions of such complexes could have therapeutic implications given the important role of Cx43 on preconditioning cardioprotection.

## Conflicts of interest

The authors confirm that there are no conflicts of interest.

## Supporting information


**Table S1** Initial list of candidates from the proteomics analysis.
