# Supplementary material for: New protein–protein interactions of mitochondrial connexin 43 in mouse heart
Source: J Cell Mol Med. 2016 Feb 25;20(5):794–803. doi: 10.1111/jcmm.12792 (PMC4831365; doi:10.1111/jcmm.12792)
Supplement: Supplementary file 1 — Table S1 Initial list of candidates from the proteomics analysis. [file JCMM-20-794-s001.doc]

| **Gene** | **UniProt** | **Protein name** | **Organism** | **Relative Diference**  **IFM-SSM** | **Present in IFM sample** | **Selected** |
| --- | --- | --- | --- | --- | --- | --- |
| Acta2 | P62737 | Actin, aortic smooth muscle | Mus musculus | 120 | No |  |
| Hspd1 | P63038 | 60 kDa heat shock protein, mitochondrial | Mus musculus | 120 | No | Yes |
| Gja1 | P23242 | Gap junction alpha-1 protein | Mus musculus | 100 | No |  |
| Myh1 | Q5SX40 | Myosin-1 | Mus musculus | 90 | No |  |
| Igg-2a | P20760 | Ig gamma-2A chain C region | Rattus norvegicus | 70 | No |  |
| Aifm1 | Q9Z0X1 | Apoptosis-inducing factor 1, mitochondrial | Mus musculus | 70 | No | Yes |
| Myl3 | P09542 | Myosin light chain 3 | Mus musculus | 60 | No |  |
| Uqcrfs1 | P20788 | Cytochrome b-c1 complex subunit Rieske, mitochondrial | Rattus norvegicus | 60 | No |  |
| Echs1 | Q8BH95 | Enoyl-CoA hydratase, mitochondrial | Mus musculus | 50 | No | Yes |
| Myl2 | P51667 | Myosin regulatory light chain 2, ventricular/cardiac muscle isoform | Mus musculus | 40 | No |  |
| Acat1 | Q8QZT1 | Acetyl-CoA acetyltransferase, mitochondrial | Mus musculus | 40 | No |  |
| Etfb | Q9DCW4 | Electron transfer flavoprotein subunit beta | Mus musculus | 40 | No | Yes |
| Aifm1 | Q9JM53 | Apoptosis-inducing factor 1, mitochondrial | Rattus norvegicus | 40 | No | Yes |
| Pdhx | Q7TQ85 | Ac1164 | Rattus norvegicus | 40 | No |  |
| Eef1a1 | P10126 | Elongation factor 1-alpha 1 | Mus musculus | 30 | No | Yes |
| Gapdh | P16858 | Glyceraldehyde-3-phosphate dehydrogenase | Mus musculus | 30 | No | Yes |
| Eci1 | P23965 | Enoyl-CoA delta isomerase 1, mitochondrial | Rattus norvegicus | 30 | No | Yes |
| Ndufs6 | P52503 | NADH dehydrogenase [ubiquinone] iron-sulfur protein 6, mitochondrial | Mus musculus | 30 | No | Yes |
| Tpm1 | P58771 | Tropomyosin alpha-1 chain | Mus musculus | 30 | No |  |
| Rps18 | P62270 | 40S ribosomal protein S18 | Mus musculus | 30 | No | Yes |
| Cycs | P62897 | Cytochrome c, somatic | Mus musculus | 30 | No | Yes |
| Hsdl2 | Q4V8F9 | Hydroxysteroid dehydrogenase-like protein 2 | Rattus norvegicus | 30 | No |  |
| Myh4 | Q5SX39 | Myosin-4 | Mus musculus | 30 | No |  |
| Cox6c | Q9CPQ1 | Cytochrome c oxidase subunit 6C | Mus musculus | 30 | No | Yes |
| Oxct1 | Q9D0K2 | Succinyl-CoA:3-ketoacid-coenzyme A transferase 1, mitochondrial | Mus musculus | 30 | No | Yes |
| Acot13 | D3ZA93 | Thioesterase superfamily member 2 | Rattus norvegicus | 30 | No |  |
| Hba1 | P01946 | Hemoglobin subunit alpha-1/2 | Rattus norvegicus | 30 | No |  |
| Hbb | P02091 | Hemoglobin subunit beta-1 | Rattus norvegicus | 20 | No |  |
| Myh7 | P02564 | Myosin-7 | Rattus norvegicus | 20 | No |  |
| Mtnd4 | P03911 | NADH-ubiquinone oxidoreductase chain 4 | Mus musculus | 20 | No |  |
| Sod2 | P07895 | Superoxide dismutase [Mn], mitochondrial | Rattus norvegicus | 20 | No | Yes |
| Acadm | P08503 | Medium-chain specific acyl-CoA dehydrogenase, mitochondrial | Rattus norvegicus | 20 | No |  |
| Sod2 | P09671 | Superoxide dismutase [Mn], mitochondrial | Mus musculus | 20 | No | Yes |
| N/A | P0C169 | Histone H2A type 1-C | Rattus norvegicus | 20 | No | Yes |
| Etfa | P13803 | Electron transfer flavoprotein subunit alpha, mitochondrial | Rattus norvegicus | 20 | No | Yes |
| Hibadh | P29266 | 3-hydroxyisobutyrate dehydrogenase, mitochondrial | Rattus norvegicus | 20 | No | Yes |
| Tfam | P40630 | Transcription factor A, mitochondrial | Mus musculus | 20 | No | Yes |
| Tnni3 | P48787 | Troponin I, cardiac muscle | Mus musculus | 20 | No |  |
| Ndufs4 | Q5XIF3 | NADH dehydrogenase [ubiquinone] iron-sulfur protein 4, mitochondrial | Rattus norvegicus | 20 | No | Yes |
| Vdac3 | Q60931 | Voltage-dependent anion-selective channel protein 3 | Mus musculus | 20 | No |  |
| Abcb7 | Q61102 | ATP-binding cassette sub-family B member 7, mitochondrial | Mus musculus | 20 | No |  |
| Etfb | Q68FU3 | Electron transfer flavoprotein subunit beta | Rattus norvegicus | 20 | No | Yes |
| Samm50 | Q6AXV4 | Sorting and assembly machinery component 50 homolog | Rattus norvegicus | 20 | No |  |
| Etfdh | Q6UPE1 | Electron transfer flavoprotein-ubiquinone oxidoreductase, mitochondrial | Rattus norvegicus | 20 | No |  |
| Actbl2 | Q8BFZ3 | Beta-actin-like protein 2 | Mus musculus | 20 | No |  |
| Dhrs4 | Q8VID1 | Dehydrogenase/reductase SDR family member 4 | Rattus norvegicus | 20 | No |  |
| Q9CPP6 | Q9CPP6 | NADH dehydrogenase [ubiquinone] 1 alpha subcomplex subunit 5 | Mus musculus | 20 | No | Yes |
| Ndufb3 | Q9CQZ6 | NADH dehydrogenase [ubiquinone] 1 beta subcomplex subunit 3 | Mus musculus | 20 | No | Yes |
| Mrps34 | Q9JIK9 | 28S ribosomal protein S34, mitochondrial | Mus musculus | 20 | No | Yes |
| Auh | Q9JLZ3 | Methylglutaconyl-CoA hydratase, mitochondrial | Mus musculus | 20 | No | Yes |
| Ogdh | Q60597 | 2-oxoglutarate dehydrogenase, mitochondrial | Mus musculus | 5 | Yes |  |
| Acat1 | P17764 | Acetyl-CoA acetyltransferase, mitochondrial | Rattus norvegicus | 4,38 | Yes |  |
| Acaa2 | P13437 | 3-ketoacyl-CoA thiolase, mitochondrial | Rattus norvegicus | 3,75 | Yes |  |
| Myh6 | Q02566 | Myosin-6 | Mus musculus | 3,59 | Yes |  |
| Pccb | Q99MN9 | Propionyl-CoA carboxylase beta chain, mitochondrial | Mus musculus | 3,44 | Yes |  |
| Chchd3 | Q9CRB9 | Coiled-coil-helix-coiled-coil-helix domain-containing protein 3, mitochondrial | Mus musculus | 3,13 | Yes |  |
| Acadvl | P50544 | Very long-chain specific acyl-CoA dehydrogenase, mitochondrial | Mus musculus | 3,13 | Yes |  |
| Acadvl | P45953 | Very long-chain specific acyl-CoA dehydrogenase, mitochondrial | Rattus norvegicus | 2,5 | Yes |  |
| Acadl | P15650 | Long-chain specific acyl-CoA dehydrogenase, mitochondrial | Rattus norvegicus | 2,5 | Yes |  |
| Prdx3 | P20108 | Thioredoxin-dependent peroxide reductase, mitochondrial | Mus musculus | 2,5 | Yes |  |
| Igh-1ª | P20761 | Ig gamma-2B chain C region | Rattus norvegicus | 2,5 | Yes |  |
| Idh3g | P70404 | Isocitrate dehydrogenase [NAD] subunit gamma 1, mitochondrial | Rattus norvegicus | 2,5 | Yes |  |
| Pdk1 | Q8BFP9 | [Pyruvate dehydrogenase [lipoamide]] kinase isozyme 1, mitochondrial | Mus musculus | 2,5 | Yes |  |
| Acad9 | Q8JZN5 | Acyl-CoA dehydrogenase family member 9, mitochondrial | Mus musculus | 2,5 | Yes |  |
| Etfdh | Q921G7 | Electron transfer flavoprotein-ubiquinone oxidoreductase, mitochondrial | Mus musculus | 2,5 | Yes |  |
| Dbt | B2GV15 | Dihydrolipoamide branched chain transacylase E2 | Rattus norvegicus | 2,5 | Yes |  |
| rCG_38845 | D3ZE15 | RCG38845, isoform CRA_b | Rattus norvegicus | 2,5 | Yes |  |
| Atp5h | P31399 | ATP synthase subunit d, mitochondrial | Rattus norvegicus | 2,08 | Yes |  |
| Vdac1 | Q60932 | Voltage-dependent anion-selective channel protein 1 | Mus musculus | 2,08 | Yes |  |
| Atp5f1 | P19511 | ATP synthase subunit b, mitochondrial | Rattus norvegicus | 1,88 | Yes |  |
| Etfa | Q99LC5 | Electron transfer flavoprotein subunit alpha, mitochondrial | Mus musculus | 1,88 | Yes |  |
| Dld | O08749 | Dihydrolipoyl dehydrogenase, mitochondrial | Mus musculus | 1,67 | Yes |  |
| Dlat | Q8BMF4 | Dihydrolipoyllysine-residue acetyltransferase component of pyruvate dehydrogenase complex | Mus musculus | 1,63 | Yes |  |
| Aldh2 | P47738 | Aldehyde dehydrogenase, mitochondrial | Mus musculus | 1,46 | Yes |  |
| Ckmt2 | Q6P8J7 | Creatine kinase S-type, mitochondrial | Mus musculus | 1,43 | Yes |  |
| Hspa9 | P38647 | Stress-70 protein, mitochondrial | Mus musculus | 1,31 | Yes |  |
| Acaa2 | Q8BWT1 | 3-ketoacyl-CoA thiolase, mitochondrial | Mus musculus | 1,25 | Yes |  |
| Immt | Q8CAQ8 | Mitochondrial inner membrane protein | Mus musculus | 1,25 | Yes |  |
| Hadhb | Q99JY0 | Trifunctional enzyme subunit beta, mitochondrial | Mus musculus | 1,25 | Yes |  |

**Table S1.** Initial list of candidates from the proteomics analysis.
